# Ferrets are valuable models for SARS-CoV-2 research

**DOI:** 10.1177/03009858211071012

**Published:** 2022-01-08

**Authors:** Malgorzata Ciurkiewicz, Federico Armando, Tom Schreiner, Nicole de Buhr, Veronika Pilchová, Vanessa Krupp-Buzimikic, Gülşah Gabriel, Maren von Köckritz-Blickwede, Wolfgang Baumgärtner, Claudia Schulz, Ingo Gerhauser

**Affiliations:** 1 University of Veterinary Medicine Hannover, Foundation, Hannover, Germany; 2 Heinrich Pette Institute, Leibniz Institute for Experimental Virology, Hamburg, Germany

**Keywords:** animal model, betacoronavirus, COVID-19, ferret, mink, pathology, SARS-CoV-1, SARS-CoV-2

## Abstract

Coronavirus disease 2019 (COVID-19), caused by severe acute respiratory syndrome coronavirus 2 (SARS-CoV-2), resulted in an ongoing pandemic with millions of deaths worldwide. Infection of humans can be asymptomatic or result in fever, fatigue, dry cough, dyspnea, and acute respiratory distress syndrome with multiorgan failure in severe cases. The pathogenesis of COVID-19 is not fully understood, and various models employing different species are currently applied. Ferrets can be infected with SARS-CoV-2 and efficiently transmit the virus to contact animals. In contrast to hamsters, ferrets usually show mild disease and viral replication restricted to the upper airways. Most reports have used the intranasal inoculation route, while the intratracheal infection model is not well characterized. Herein, we present clinical, virological, and pathological data from young ferrets intratracheally inoculated with SARS-CoV-2. Infected animals showed no significant clinical signs, and had transient infection with peak viral RNA loads at 4 days postinfection, mild to moderate rhinitis, and pulmonary endothelialitis/vasculitis. Viral antigen was exclusively found in the respiratory epithelium of the nasal cavity, indicating a particular tropism for cells in this location. Viral antigen was associated with epithelial damage and influx of inflammatory cells, including activated neutrophils releasing neutrophil extracellular traps. Scanning electron microscopy of the nasal respiratory mucosa revealed loss of cilia, shedding, and rupture of epithelial cells. The currently established ferret SARS-CoV-2 infection models are comparatively discussed with SARS-CoV-2 pathogenesis in mink, and the advantages and disadvantages of both species as research models for zoonotic betacoronaviruses are highlighted.

The devastating pandemic caused by the severe acute respiratory syndrome coronavirus 2 (SARS-CoV-2) prompted the use of animal research models including hamsters, mice, nonhuman primates, and ferrets to investigate clinical pathology, and prophylactic and therapeutic measures for humans *in vivo*.^12,26^ Domestic ferrets (*Mustela putorius furo*) and American mink (*Neovison vison*) are members of the *Mustelidae* family.^55^ These 2 mustelids are susceptible to different alphacoronavirus and betacoronavirus infections including ferret enteric coronavirus, ferret systemic coronavirus, mink epizootic catarrhal gastroenteritis, and SARS-CoV-2.^27,55^ Ferrets have been used as a model organism to study influenza A and SARS-CoV-1 infection,^5,59^ whereas mink have been bred and raised mainly in the fur farming industry.^16^ Ferrets have several advantages to other laboratory animals as they (1) are naturally susceptible to various human and animal pathogens of the respiratory tract and central nervous system, (2) frequently reproduce clinical and pathologic characteristics of human infectious diseases, such as influenza, (3) have a lung physiology similar to humans, and (4) are considerably larger and have a longer live span (7-10 years) than other laboratory animals.^14^ In addition, the genetic variability in ferrets commercially available for research purposes is higher compared to inbred mouse strains, which has certain advantages for modeling possible outcomes of viral infection. During the SARS-CoV-1 outbreak in 2003, ferrets were found to be a valuable model for the development of vaccines and antiviral strategies because they are naturally susceptible to the virus and show efficient replication and shedding.^11,13,58^ Similarly, ferrets can be successfully infected with SARS-CoV-2 and efficiently transmit the virus to contact animals.^23,44^ Severe acute respiratory syndrome coronavirus 1- and SARS-CoV-2-induced clinical signs and lesions may vary and are generally mild in ferrets.^23,48,58^ Nevertheless, aged ferrets can develop high viral loads with longer nasal virus shedding, as well as severe pneumonia and clinical signs similar to human patients suffering from coronavirus disease 2019 (COVID-19).^24^ In this study, we present data of SARS-CoV-2-infected ferrets and review the current literature about natural and experimental betacoronavirus infections in mustelids to evaluate their value as translational animal models to study different aspects of respiratory viral infections.

## Material and Methods

### Animal Experiment

Twelve 8-month-old healthy specific pathogen-free female ferrets (F1-F12) were purchased at a commercial breeder (Triple F Farms, USA) and kept in groups of 3 animals (according to day of euthanasia) in open cages (ferret suite, Tecniplast) in the biosafety level-3 facility of the Research Center for Emerging Infections and Zoonoses (RIZ), University of Veterinary Medicine Hannover, Foundation, Germany, according to the Directive 2010/63/EU. The animals were randomly selected by the breeder. Before the experiment, all animals were confirmed by virological (see below) and serological analyses (Friedrich-Loeffler-Institute, Isle of Riems) as free of SARS-CoV-2 infection. Health monitoring of the ferrets revealed that the animals were free of infection with various respiratory bacterial and viral pathogens including *Bordetella bronchiseptica, Streptococcus pneumoniae*, rotavirus, and Aleutian disease virus, while ferret enteric coronavirus and cryptosporidia were detected in pooled fecal samples of the ferrets by a veterinary diagnostic laboratory (IDEXX). The ferret colony of the commercial breeder was regularly tested free of *Helicobacter mustelae*.

Three control ferrets (F1-F3) were neither infected with a virus nor did they receive any sort of vehicle and were euthanized 2 days before infection of F4 to F12. Nine ferrets (F4–F12) were infected intratracheally with 1 mL of 106 TCID50 of SARS-CoV-2 using the human 2019-nCoV isolate (Ref-SKU: 026V-03883 strain BavPat1/2020, kindly provided by Christian Drosten, Charité, Berlin, Germany) passaged once on Vero E6 cells (kind gift from Bart L. Haagmans, Erasmus MC, Rotterdam, The Netherlands) in Dulbecco′s Modified Eagle′s Medium (Gibco) with 1% penicillin/streptomycin (Sigma), 1% GlutaMAX (Gibco) and 2% fetal bovine serum (Bio&Sell). The ferrets were monitored daily for clinical signs, body weight, and rectal body temperature (Supplemental Table S1). For virological analyses, oropharyngeal and fecal swabs were taken 3 days before SARS-CoV-2 infection and at 2, 4, 5, 7, 9, 12, 14, 17, and 21 days postinfection (dpi). In addition, blood samples were collected before infection (F1-F12) and from a group of 3 animals on their day of euthanasia: at 4 dpi (F4-F6), 7 dpi (F7-F9), and 21 dpi (F10-F12). Blood samples were also collected at 14 dpi from ferrets F10 to F12 (Supplemental Table S2). At the experimental endpoints (2 days before infection for controls; 4, 7, or 21 days after infection for SARS-CoV-2 infected) groups of 3 ferrets were put under anesthesia (10 mg/kg ketamine, 0.5 mg/kg midazolam, 0.1 mg/kg medetomidin) and euthanized by exsanguination (incision in the aorta and caudal vena cava). Bronchoalveolar lavage fluid (BALF) was obtained in sternal recumbency immediately after euthanasia. An 11-cm catheter was inserted into the trachea using a laryngoscope, 3 mL phosphate-buffered saline (PBS) with 0.2% bovine serum albumin (BSA) and 1% penicillin/streptomycin were injected using a 5 mL syringe, the body was slightly turned and approximately 1.5 mL were recovered by aspiration. Then, a full necropsy was performed, lung weight was obtained, and tissue samples were taken for further analysis (Supplemental Table S2). The animal experiment including the described experimental protocol were approved and authorized by local authorities, Niedersächsisches Landesamt für Verbraucherschutz- und Lebensmittelsicherheit (LAVES), Oldenburg, Germany, permission number: 33.19-42502-04-20/3402. All animal procedures were performed in accordance with the German regulations and legal requirements.

### Virological Analysis

Nucleic acid was extracted from 100 μL of swab, ethylenediaminetetraacetic acid (EDTA) whole-blood and BALF samples and tissue homogenate supernatant with Nucleo MagVet Kit (Macherey-Nagel) with a KingFisher 96 platform (Thermo Fisher Scientific) and eluted in 100 µL. Real-time reverse transcription polymerase chain reaction (RT-qPCR) was conducted as described previously (Supplemental Table S3).^20,22^ Briefly, 2.5 µL template was amplified using the AgPath-ID™ One-Step RT-PCR Reagents (Thermo Fisher Scientific) with SARS-CoV-2-specific primers and probes targeting the RdRp gene of SARS-CoV-2 (SARS-2-IP4 assay of Institute Pasteur, recommended by the WHO) and including an internal control system at a CFX96 Touch Real-Time PCR Detection System (Bio-Rad), quantification cycle (Cq) values <40 were considered positive.^20,22^ Severe acute respiratory syndrome coronavirus-2 RNA was quantified using *in vitro* transcribed RNA derived from strain BetaCoV_Wuhan_WIV04_2019 (EPI_ISL_402124) kindly provided by Institute Pasteur as standard. The transcript contains the amplification regions of the RdRp (“IP2 and IP4”) and E gene as positive strand.

### Histology and Immunohistochemistry

Two- to 3-µm sections of formalin-fixed and paraffin-embedded tissues were routinely stained with hematoxylin and eosin (HE). Microscopic examination was conducted by a board-certified veterinary pathologist (IG). For immunohistochemistry, dewaxed and rehydrated sections were incubated in ethanol with 0.5% hydrogen peroxide for 30 min to block endogenous peroxidase. To detect SARS-CoV-2 and feline infectious peritonitis (FIP) virus, antigen retrieval was performed by boiling the slides for 20 min in citrate-EDTA buffer (10 mM citrate acid, 2 mM EDTA, 0.05% Tween 20%, pH 6.2) or in citrate buffer (10 mM citrate acid, pH 6.0) using a microwave oven, respectively. Subsequently, sections were incubated with mouse monoclonal antibodies directed against SARS-CoV-2 nucleocapsid (40143-MM05; Sino Biological Europe; 1:3200 in PBS with 1% BSA and 0.3% TritonX-100) or FIP virus (FIPV3-70; Custom Monoclonals International; 1:10,000 in PBS with 1% BSA) for 1 h at room temperature. Negative control sections were incubated with ascites of Balb/c mice instead of primary antibodies. Lung of a SARS-CoV-2-infected hamster and liver of a cat with FIP were used as positive tissue controls. Immunolabeling was visualized by the Dako EnVision+ System (K4001; Agilent Technologies) or a goat-anti-mouse secondary antibody (BA-9200; Vector Laboratories; 1:200) and the avidin-biotin-peroxidase-complex (ABC) method (PK6100; Vector Laboratories) with 3,3-diaminobenzidine (DAB) as substrate, respectively. Finally, sections were slightly counterstained with Mayer’s hematoxylin. Photos of stained sections were generated by a digital microscope (HS All-in-one Fluorescence Microscope BZ-9000 Generation II; HS All-in-one Fluorescence Microscope BZ-II Analyzer, BIOREVO, KEYENCE).

### NET Examination

For neutrophil extracellular trap (NET) detection, paraffin sections from animals at 4 dpi (serial cuts to HE and virus antigen staining) were analyzed. The immunofluorescence staining of paraffin section was performed as previously described.^4^ Briefly, first mouse IgG2a anti-DNA/histone (MAB3864, Millipore; 0.55 mg; 1:500) and rabbit anti-human myeloperoxidase antibodies (A0398, Dako; 3.2 mg, 1:300) in blocking buffer were incubated overnight at 4°C. As secondary antibodies, goat anti-mouse antibody (Alexa488PLUS, Thermo Fisher Scientific) and goat anti-rabbit antibody (Alexa568, Thermo Fisher Scientific) were diluted 1:500 in blocking buffer. Nuclei were stained with Hoechst 33342 (Sigma). Samples were recorded using a Leica TCS SP5 AOBS confocal inverted-base fluorescence microscope with HCX PL APO 40 × 0.75–1.25 oil immersion objective. The settings were adjusted using isotype control antibodies in separate preparations. Representative three-dimensional (3D) images of *z*-stacks were constructed with LAS X 3D Version 3.1.0 software (Leica).

### Scanning Electron Microscopy

At necropsy, samples from the nasal cavity (cranial and caudal conchae, septum) were collected for scanning electron microscopy (SEM). Samples were fixed in 5% glutaraldehyde buffered with 0.1 M cacodylate buffer (Serva Electrophoresis) and were subsequently embedded by a modified osmium (O)-thiocarbohydrazide (T)-embedding (OTOTO) protocol, followed by critical-point-drying and coating with gold in a sputter-coater (SCD040, Oerlikon Balzers), as described previously.^10^ Embedded samples were mounted on 0,5” Aluminum Specimen Stubs (Agar Scientific) using 12 mm Leit-Tabs (Plano) and Conductive Carbon Cement after Göcke (Plano) and examined using a Zeiss EVO 15 scanning electron microscope (Carl Zeiss Microscopy) operating with 10 kV.

## Results

### Clinical Evaluation and Virological Results

**Figure 1.**
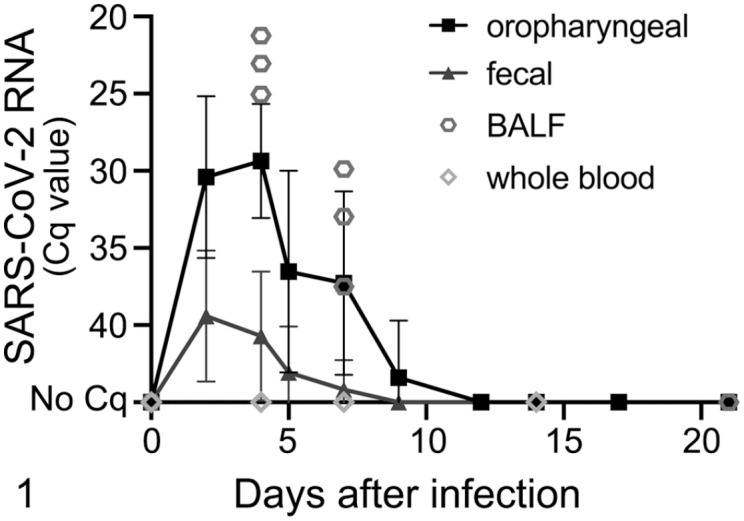
Progression of viral RNA loads in ferret samples after intratracheal infection with SARS-CoV-2 up to 21 days postinfection (dpi). Bronchoalveolar lavage fluid (BALF) and whole blood were collected after euthanasia at 4, 7, and 21 dpi (3 animals per timepoint). Oropharyngeal (OP) and fecal (FE) swabs were collected at 2, 4, 5, 7, 9, 12, 14, 17, and 21 dpi. SARS-CoV-2-RNA loads peaked in oropharyngeal swabs (quantification cycle [Cq] 21.06) and BALF (Cq 21.24) at 4 dpi. Fecal swab viral RNA loads were generally lower (Cq 31.50) and peaked at 2 dpi. SARS-CoV-2 RNA was detected up to 7 dpi in fecal swabs and BALF and up to 9 dpi in oropharyngeal swabs in individual animals. SARS-CoV-2 RNA was not detected in whole blood samples. Data show mean ± standard deviation. SARS-CoV-2, severe acute respiratory syndrome coronavirus 2.

**Figure 2.**
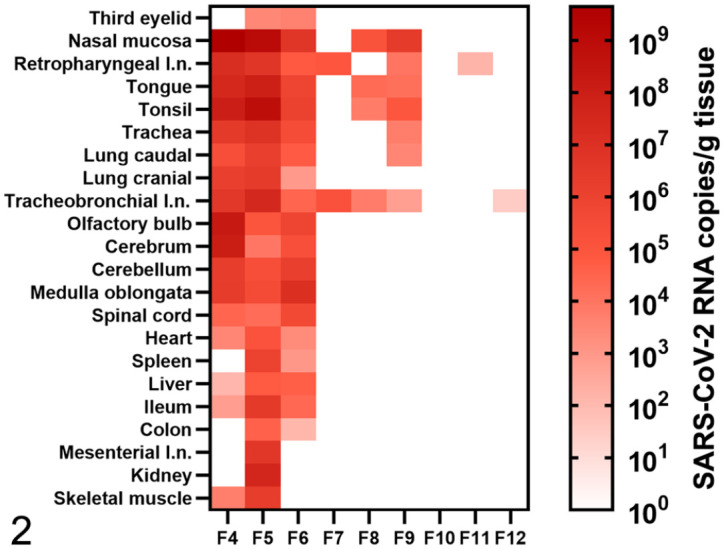
SARS-CoV-2 RNA copies/g in tissues of ferrets intratracheally infected with SARS-CoV-2, euthanized at 4 days postinfection (dpi, F4-F6), 7 dpi (F7-F9) or 21 dpi (F10-F12). SARS-CoV-2 RNA was detected in all analyzed tissue samples at 4 dpi with peak viral RNA loads in the nasal mucosa or medulla oblongata depending on the animal. After 7 dpi, 7 of 8 tissues of the upper and lower respiratory tract or associated lymphoid tissue were PCR-positive in individual ferrets. At 21 dpi, viral RNA was only detected in the retropharyngeal or tracheobronchial lymph node (l. n.) of 1 ferret each. SARS-CoV-2, severe acute respiratory syndrome coronavirus 2.

An overview of the clinical and virological results is given in Figs. 1 and 2, and Supplemental Tables S4 to S6. Generally, no clinical signs were observed in infected ferrets, except for poor general condition in 1 animal at 1 dpi and mild transient weight loss (<10%) in 2 animals at 2 dpi. Fecal and oropharyngeal swab samples collected during the experiment were SARS-CoV-2 RNA-positive up to 9 dpi with individual variations (Fig. 1). The highest viral load was detected in oropharyngeal swabs at 4 dpi (Fig. 1). BALF samples, collected at the experimental endpoints, were SARS-CoV-2 RNA-positive at 4 and 7 dpi, but negative at 21 dpi (Fig. 1). Whole-blood samples were consistently negative (Fig. 1). Severe acute respiratory syndrome coronavirus-2 RNA was found in a broad range of tissue samples collected at 4 dpi, with highest loads in the nasal mucosa or medulla oblongata (Fig. 2). At 7 dpi, viral RNA was found in a few tissue samples from the respiratory tract and associated lymphoid tissues (Fig. 2). At 21 dpi, low SARS-CoV-2-RNA loads were still found in the retropharyngeal lymph node or the bronchial lymph node in 1 animal each (Fig. 2). Severe acute respiratory syndrome coronavirus-2 RNA was not detected in any sample from control animals.

In summary, infected ferrets showed no significant clinical signs, but a transient broad tissue distribution of viral RNA. The highest viral loads were found in the respiratory tract and associated lymphoid tissues in early infection.

### Macroscopic and Microscopic Findings

No macroscopic lesions were found in the respiratory tract of any of the ferrets (Supplemental Figs. S1-S4). Individual ferrets had higher lung weights at 4 dpi (F5, F6) compared to controls. However, there were large variations of the lung weights in the respective groups, possibly due to residual fluid from BALF sampling, and no statistically significant differences between groups were detected (Supplemental Fig. S5).

**Figure 3–6.**
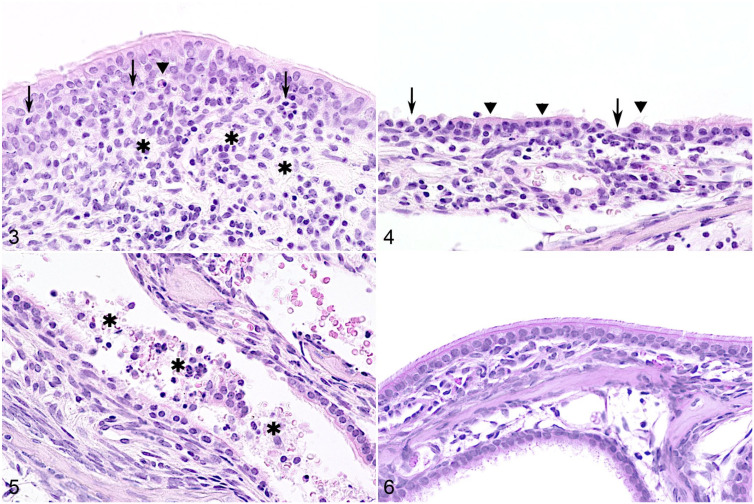
Severe acute respiratory syndrome coronavirus 2 infection, rostral nasal concha, and ferret. Hematoxylin and eosin (HE). **Figure 3.** Ferret F5, 4 days postinfection (dpi). Lymphocytes, macrophages, and a few neutrophils are present in the lamina propria (asterisks) and the respiratory epithelium (arrows). Rarely, there is single-cell necrosis of epithelial cells (arrowhead). **Figure 4.** Ferret F6, 4 dpi. The respiratory epithelium is attenuated and has partial or complete loss of cilia (arrowheads) and erosions (arrows). **Figure 5.** Ferret F6, 4 dpi. Intraluminal aggregates of cellular debris, desquamated epithelial cells, and neutrophils (asterisks). **Figure 6.** Normal, rostral nasal concha, control ferret F1. The mucosa has ciliated epithelium with rare goblet cells. The lamina propria occasionally contains individual leukocytes.

Histopathology findings are summarized in Supplemental Tables S7 and S8. Mild to moderate multifocal lymphohistiocytic and neutrophilic rhinitis with rare single-cell necrosis in the overlying epithelium was found in infected ferrets (Fig. 3). More frequently, the epithelium was attenuated, desquamated, and eroded with loss of cilia (Fig. 4). The lumen contained small amounts of mucus, cellular debris, and neutrophils (Fig. 5). The lesions were restricted to the respiratory epithelium in the cranial conchae, while no alterations were detected in the olfactory mucosa. In the trachea, ferrets euthanized at 4 dpi showed similar inflammatory lesions also including eosinophils and degenerative epithelial changes. No lesions were found in the nasal conchae or trachea of control ferrets (Fig. 6).

**Figures 7–12.**
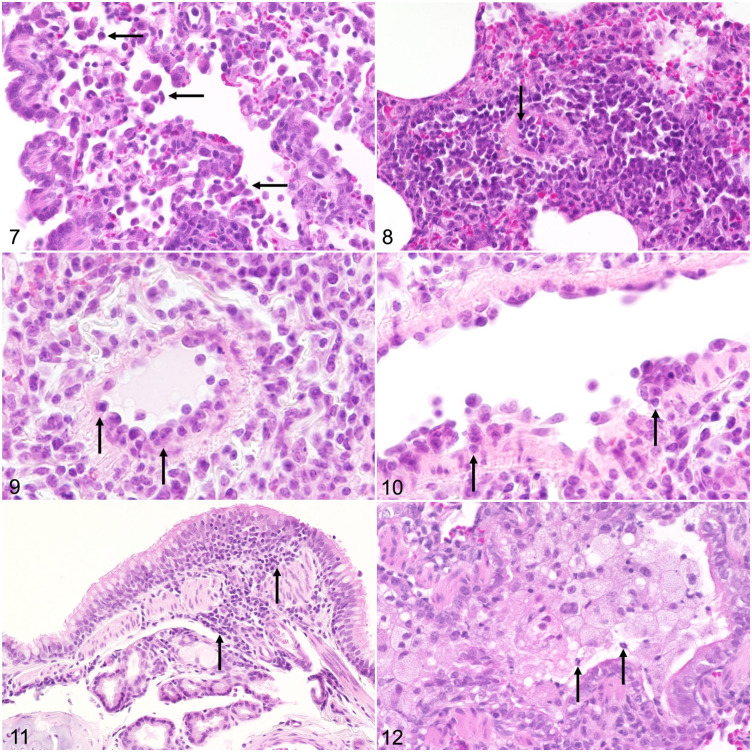
Severe acute respiratory syndrome coronavirus 2 infection, lung, and ferret. Hematoxylin and eosin (HE). **Figure 7.** Ferret F7, 7 dpi. Mild alveolar histiocytosis (arrows). **Figure 8.** Ferret F11, 21 dpi. Vasculitis, with lymphocytes and macrophages infiltrating the intima (endothelialitis, arrow) and the perivascular interstitium. **Figure 9.** Ferret F7, 7 dpi. Endothelialitis, with hypertrophy of endothelial cells (arrows). **Figure 10.** Ferret F7, 7 dpi. Endothelialitis, with attachment of lymphocytes and macrophages to endothelial cells and subsequent migration into the subendothelial tissue (arrows). **Figure 11.** Ferret F11, 21 dpi. Bronchitis. There is infiltration of the lamina propria by lymphocytes and macrophages (arrows). **Figure 12.** Ferret F8, 7 dpi. Bronchiolitis, with abundant vacuolated macrophages and few neutrophils in the lumen (arrows). Mild inflammatory cell infiltrates are also present in the mucosa.

In the lung, mild alveolar histiocytosis was found in 3 individual infected ferrets euthanized at 4, 7, and 21 dpi (Fig. 7). One animal euthanized at 4 dpi additionally showed mild suppurative pneumonia. Alveolar edema, necrosis of alveolar septa, or syncytia were not detected in any ferret. All animals with alveolar lesions also showed mild to moderate vascular lesions, characterized by lymphohistiocytic endothelialitis and perivasculitis with endothelial hypertrophy of mostly small-sized vessels (Figs. 8–10). Mild BALT hyperplasia was found in the lung of all infected ferrets at 4 and 7 dpi and 1 ferret at 21 dpi but not in control ferrets. Bronchial and bronchiolar inflammation was observed in infected as well as control ferrets but was more severe in some infected animals (Figs. 11 and 12). The lesions were characterized by infiltration of the bronchial and bronchiolar mucosa with lymphocytes, plasma cells, macrophages, eosinophils (7/12), and neutrophils (2/12), which were also occasionally present in the lumen.

Inflammatory lesions, which most likely were not related to SARS-CoV-2 infection, were common in the gastrointestinal tract and liver of control and infected ferrets (Supplemental Figs. S6–S9). Additional background lesions observed in individual ferrets included mild granulomatous or lymphohistiocytic meningitis, (Supplemental Fig. S10), moderate lymphohistiocytic inflammation adjacent to the aorta associated with a mild endothelialitis (Supplemental Fig. S11), lymphohistiocytic conjunctivitis, lymphoplasmahistiocytic interstitial nephritis, demodicosis, lymphohistiocytic sialoadenitis (Supplemental Fig. S12), lymphohistiocytic and suppurative pharyngitis, lymphohistiocytic endocarditis and myocarditis (Supplemental Fig. S13), and renal cysts. The background lesions are listed in Supplemental Table S8.

In summary, SARS-CoV-2-infected ferrets showed rhinitis and tracheitis associated with epithelial damage. Additional lesions found only in infected ferrets were mild interstitial or suppurative pneumonia with alveolar histiocytosis and BALT hyperplasia, as well as moderate lymphohistiocytic endothelialitis and perivasculitis of small-sized vessels. Bronchial and bronchiolar inflammation was found in infected and control animals, and therefore could not solely be attributed to SARS-CoV-2 infection. Inflammatory background lesions in various other organs were common in ferrets regardless of their infection status.

### Immunohistochemistry and Immunofluorescence

**Figures 13–16.**
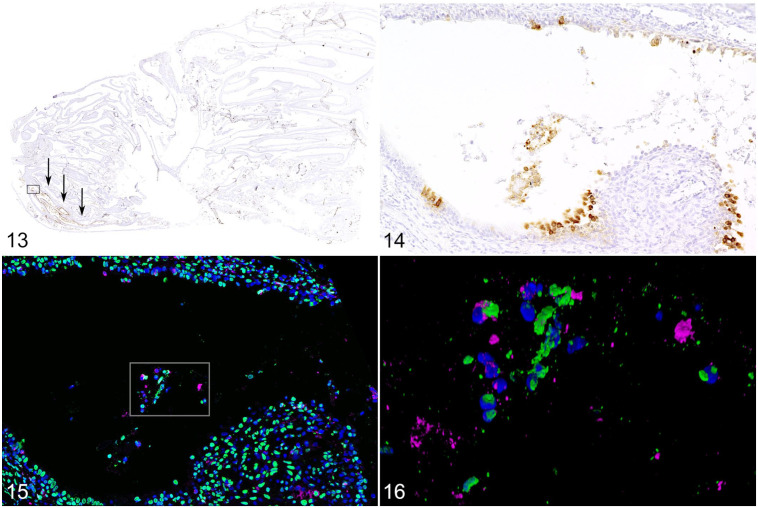
SARS-CoV-2 infection, nasal conchae, ferret F6, 4 days postinfection. Immunohistochemistry. **Figure 13.** Positive signal for SARS-CoV-2 nucleoprotein is only detected in the respiratory mucosa of the rostral conchae (arrows) and not in other areas of the nasal conchae. **Figure 14.** Higher magnification of area enclosed by the rectangle in Fig. 13, showing positive signal in intact and desquamated epithelial cells. **Figures 15–16.** Neutrophils release neutrophil extracellular traps (NETs) in nasal conchae. The specimen is a serial section immediately consecutive to that depicted in Fig. 13; Figure 16 represents the box shown in Fig. 15. Double-labeling for myeloperoxidase (MPO, magenta) and DNA-histone complexes (green); nuclei are stained blue (Hoechst 33342). There is intraluminal accumulation of desquamated cells and neutrophils (corresponding to the box with virus-positive cells in Fig. 13), and MPO (magenta) and DNA-histone (green) are in the extracellular space directly adjacent to neutrophils, indicative of degranulation and formation of NETs. Figure 15 is a 3D-reconstruction of confocal laser images (54 *z*-stack frames; 8.9 µm total size; approx. 0.17-µm steps). SARS-CoV-2, severe acute respiratory syndrome coronavirus 2.

Severe acute respiratory syndrome coronavirus 2 nucleoprotein (NP) was exclusively found in respiratory epithelium in the rostral nasal conchae of all ferrets sacrificed at 4 dpi (Figs. 13 and 14), whereas immunopositive cells were absent at later time points. Positive signals were detected in a small to moderate number of cells and had a diffuse cytoplasmic distribution. No positive cells were detected in the olfactory epithelium. Moreover, SARS-CoV-2 NP antigen was not detected in any other organ including the meningeal and aortic inflammatory lesions. An absence of immunolabelling with the antibody clone FIPV3-70 excluded infection of brain, aorta, heart, lung, and salivary glands with ferret coronavirus, FIP virus type 1 and 2, canine coronavirus, pig coronavirus transmissible gastroenteritis virus, or bovine coronavirus.

Neutrophil extracellular traps are suspected to contribute to tissue damage in human COVID-19 and NETs are also released by neutrophils in SARS-CoV-2 infected hamsters.^1,4^ Therefore, we analyzed the virus-positive lesions in the nasal conchae for NET-formation by dual immunolabeling for myeloperoxidase and DNA-histone-1 complexes. Myeloperoxidase and DNA-histone material was found adjacent to activated neutrophils indicating degranulation and formation of NETs (Figs. 15 and 16). Since viral antigen was only found in the nasal cavity, other tissues were not investigated for NET formation.

In summary, SARS-CoV-2 infection was transient, mild, and mostly restricted to the upper respiratory tract. The virus appeared to specifically target respiratory epithelium in the nasal cavity in this intratracheal infection model and infection was associated with shedding of epithelial cells and formation of NETs.

### Scanning Electron Microscopy

**Figures 17–20.**
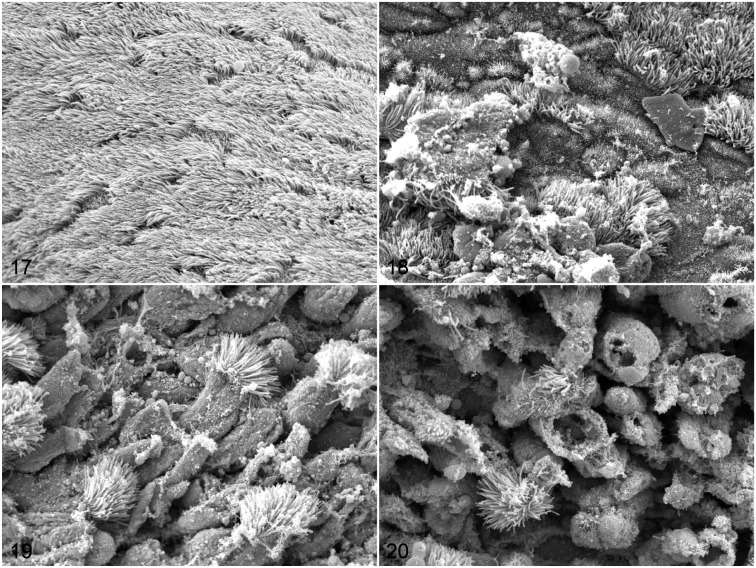
Mock-infected (Fig. 17) or severe acute respiratory syndrome coronavirus 2-infected (Figs. 18–20) ferrets, 4 days postinfection, nasal respiratory epithelium. Scanning electron microscopy. **Figure 17.** Mock-infected ferret F1. Intact epithelium is completely covered by cilia. **Figure 18.** Infected ferret F4. Patchy loss of cilia on cells that appear otherwise intact. **Figure 19.** Infected ferret F6. Detachment of ciliated cells. **Figure 20.** Infected ferret F5. Rupture and detachment of epithelial cells. Scale bars: 5 µm.

In mock-infected ferrets, the respiratory mucosa was completely covered by cilia arranged in parallel along the apical surface (Fig. 17). In all SARS-CoV-2-infected ferrets sacrificed at 4 dpi, the respiratory mucosa showed multifocal loss of cilia from cells that appeared otherwise intact, with increased amounts of mucus and cellular debris on the epithelial surface (Fig. 18). Remaining cilia occasionally showed disordered polarity and shortening but no other abnormalities. In other areas, detachment, shedding, and rupture of ciliated cells were observed (Figs. 19 and 20). Overall, the lesions had a patchy distribution and affected only a small part of the surface. Thus, ultrastructural analysis revealed only mild lesions in the nasal mucosa of SARS-CoV-2-infected ferrets, which correlated with the microscopic findings.

## Discussion

Ferrets are established animal models to study the pathogenesis of respiratory viral infections such as influenza A virus, respiratory syncytial virus and SARS-CoV-1.^14,58,59^ The ferret model has been extensively investigated to evaluate its potential to mimic the pathogenesis of COVID-19 in humans and for further research on transmission, prophylaxis, and therapy (Supplemental Table S9).^25^ Clinical signs of SARS-CoV-2 infection are either absent^49^ or mild^23,35,48,52^ in intranasally infected ferrets and include fever, loss of appetite, lethargy, ruffled fur, coughing, and “snoring.” Intratracheal infection has only been described in one previous study that did not report detailed results of the clinical monitoring, although severe disease and death were not observed.^52^ Highest levels of viral RNA are present in the upper respiratory tract and peak at 4 to 7 dpi^15,48^ but viral RNA was also found in lung, gastrointestinal tract, liver, spleen, kidney, and brain.^23,35,49^ Nevertheless, infectious virus was only detected in the upper respiratory tract, whereas the lung seems to contain only inhaled viral particles.^35,48,62^ In our study, no viral RNA was detected in whole-blood samples despite wide dissemination of SARS-CoV-2 to various organs suggesting transient viremia soon after infection. Viral RNA was readily detected in the respiratory and intestinal tract and draining lymph nodes at 4 dpi and the retropharyngeal or bronchial lymph node was still PCR-positive in individual ferrets at 21 dpi. Nevertheless, SARS-CoV-2 RNA shedding ceased after the onset of the humoral immune response between 7 and 10 dpi and virus antigen was only detected in the nasal cavity at 4 dpi similar to previous reports.^15,23,35,48,49^ However, some studies also reported scattered cells containing SARS-CoV-2 RNA or antigen in the larynx, trachea, lung, and intestine.^23,35,48^ Interestingly, in the nasal cavity, viral antigen and associated lesions were restricted to the respiratory mucosa in this study, whereas previous ferret studies also reported infected cells in the olfactory epithelium.^15,49^ This discrepancy could be due to differences in the virus isolate, infection dose or other factors. The selective detection of viral antigen in the cranial conchae could reflect stronger tropism of the virus to the ciliated respiratory epithelia. In the ferret model of influenza A virus infection, preferential infection of respiratory epithelium is observed in viruses that are efficiently transmitted via air, in contrast to the nonairborne transmissible strain H5N1, which does not efficiently infect nasal ciliated epithelia.^45^ A stronger tropism for respiratory mucosa could also be a driver for the very efficient virus transmission seen in ferrets and humans infected with SARS-CoV-2.^44^ This hypothesis could be substantiated by further experiments, for instance by infection of primary mucosal explants derived from different regions of the nasal cavity.^47^

Histological lesions associated with SARS-CoV-2 infection in young ferrets are usually mild and gross lesions are generally absent (Supplemental Table S9). In most cases, mild rhinitis with epithelial necrosis, mild bronchopneumonia, and mild interstitial pneumonia with BALT and type II pneumocyte hyperplasia and perivascular and peribronchiolar cuffing have been described.^35,48,49^ Moreover, one study detected severe vasculitis and perivasculitis at 13 dpi.^52^ In the present study, lesions found in the nasal conchae and trachea consisted of patchy inflammation along with limited epithelial damage. The lesions in the nasal cavity were associated with viral antigen, while no antigen was found in the trachea. Therefore, the authors cannot exclude that the tracheal lesions were caused by trauma secondary to intratracheal inoculation. Severe acute respiratory syndrome coronavirus-2-induced damage and loss of cilia, and downregulation of genes associated with ciliogenesis and ciliary function has been described *in vitro* and *in vivo*.^19,39,43,66^ Dysfunctional mucociliary clearance predisposes to secondary pulmonary infections, which occur in nearly 35% of severe cases of COVID-19 and worsen the outcome.^57^ The exact mode of ciliary damage remains undetermined and requires further examination. Our results show that the ferret could be a useful model to study this particular aspect of SARS-CoV-2 infection.

In contrast to the nasal cavity, we could not demonstrate lesions clearly associated with viral infection in the lower respiratory tract. Despite a positive PCR result, viral antigen was not detected in the lungs of the ferrets, which could be due to viral loads below the detection limit of immunohistochemistry or due to rapid pulmonary virus clearance. In contrast to human patients, nonhuman primate and mouse models of COVID-19, and naturally infected mink, the ferrets of this study did not show any syncytia or evidence of alveolar damage in lung.^6,8,34,40,46,54,64^ The main histological findings in the lungs were bronchiolitis, alveolar histiocytosis, and BALT hyperplasia as well as vascular lesions. Bronchiolitis was also found in control animals and thus cannot be solely attributed to infection. Alveolar histiocytosis and BALT hyperplasia were only found in infected animals, but these lesions are common findings in ferrets and therefore most likely represent background lesions. The most striking pulmonary findings detected exclusively in infected animals were vascular lesions, which were characterized by mild infiltration of the intima with lymphocytes and macrophages (endothelialitis). To the authors’ knowledge, similar lesions have not been described in the ferret COVID-19 model so far. Vasculitis and endothelialitis associated with breakdown of the vascular barrier and subsequent loss of fluids have also recently been described in Syrian golden hamsters infected with SARS-CoV-2.^3,4^ Moreover, vascular inflammation with edema and thrombus formation represents a characteristic feature of severe COVID-19 cases in humans.^2,56,60^ Further studies are needed to unravel the pathologic mechanisms and functional consequences of these vascular changes in SARS-CoV-2-infected ferrets.

Our findings are generally in line with previous clinical, virological, and pathological findings including mild clinical signs,^15^ a peak in viral RNA at 4 dpi, and mild pulmonary lesions.^15,35,48^ Therefore, the intratracheal inoculation route seems to offer no advantage to intranasal infection with regard to severity of induced pulmonary infection or lesions. This finding is in contrast to other ferret models of respiratory viral infections. For instance, in the ferret model of H5N1 influenza infection, intratracheal infection leads to severe bronchointerstitial pneumonia, while intranasal infection is less efficient in causing pulmonary lesions.^7^ The primary infection site does not seem to influence disease severity in SARS-CoV-2 infected ferrets, indicating that the lower respiratory tract in this species is equipped with host factors that specifically limit infection and/or replication of this particular virus. Interestingly, more extensive pulmonary inflammation with up to 50% of lung involvement and more severe clinical signs have been described in aged ferrets (in a preprint).^24^

NET-formation was identified in many animal species including mice, rats, chinchilla, and ferrets and described as one pathogenic trigger in severe COVID-19.^4,36,37^ During formation of NETs (i.e. the process of NETosis), myeloperoxidase translocates from the neutrophil granule to the nucleus and promotes nuclear decondensation.^42^ The associated relaxation of chromatin during NETosis results in the exposure of the epitopes for antichromatin (DNA-histone-complex) antibodies, which are not detectable in normal lobulated nuclei of neutrophils.^9^ Thus, using an antibody against DNA-histone-complexes, different stages of NET-formation can be identified based on characteristic morphological changes of the nucleus upon stimulation of NETs.^61^ Disintegration of the nuclear membrane occurs concomitantly with cytoplasmic granule dissolution, allowing NET components to mix in the cytoplasm and release to the extracellular environment. We showed infiltration of activated neutrophils in the nasal cavity of SARS-CoV-2-infected ferrets, which was closely associated with viral antigen. These activated neutrophils were surrounded by myeloperoxidase and also DNA-histone material indicating degranulation and NET formation as previously demonstrated in lungs of SARS-CoV-2-infected Syrian golden hamsters.^4^ However, it is unclear if NETs are detrimental or beneficial during SARS-CoV-2 infection. It can be hypothesized that NETs help to control SARS-CoV-2 infection until detrimental effects become predominant in some hosts due to unknown circumstances. Neutrophil extracellular trap**s** can immobilize enveloped viruses due to the attachment of the positively charged histones from NETs to the negatively charged viral envelope.^50^ Furthermore, antimicrobial peptides are associated with NETs^38^ and can act against viruses including SARS-CoV-2.^17^ Further studies are needed to understand if beneficial NET-formation could explain the low virus load, few histological lesions, and milder clinical signs in ferrets.

The endocarditis, meningitis, and sialoadenitis found in individual ferrets are most likely incidental findings but immune-mediated mechanisms triggered by SARS-CoV-2 infection cannot be ruled out completely. Similarly, gastrointestinal and hepatic inflammation found in this study represents a common background lesion in ferrets, which is most likely not related to SARS-CoV-2 infection.^21,29^ Subclinical infection with *Cryptosporidium* sp. and ferret enteric coronavirus detected by routine health monitoring may also explain intestinal inflammation. *Demodex* sp. were described in another study in 9 out of 25 animals affecting hair follicles and sebaceous glands of the perianal, vulvar, preputial, facial, and caudal abdominal skin.^32^

Ferrets have been used previously as a model species for SARS-CoV-1 infection with some differences in the clinical and pathological picture compared to SARS-CoV-2. Animals infected with SARS-CoV-1 showed fever, sneezing, and sometimes diarrhea^11^ or developed lethargy (Supplemental Table S10).^58^ Interestingly, vaccination with recombinant vaccinia virus Ankara (MVA) expressing the SARS-CoV-1 spike protein did not prevent virus replication and even enhanced nonsuppurative hepatitis possibly by antibody-dependent immune mechanisms.^13^ Lung lesions included mild bronchointerstitial pneumonia with type-II pneumocyte hyperplasia and marked perivascular and peribronchiolar mononuclear cell infiltrates. Bronchial lumina also contained mucus and neutrophils.^11^ Gross lesions were characterized by multifocal pulmonary consolidation, dark red and enlarged mesenteric lymph nodes, dark red friable liver, and mottled red-and-pink spleen.^58^ Thus, SARS-CoV-1 produces more severe disease in ferrets compared to SARS-CoV-2. Moreover, the 2 viruses show different cell tropisms in ferrets. Severe acute respiratory syndrome coronavirus-1 antigen was detected in type-I and -II pneumocytes and alveolar macrophages,^58^ while SARS-CoV-2 predominantly localizes to the upper respiratory tract. Angiotensin-converting enzyme 2 (ACE2) represents the major cell entry receptor for SARS-CoV-1 and SARS-CoV-2^30,51^ and ACE2 mRNA can be detected in the lung, heart, spleen, and small intestine of ferrets.^63^ The cellular localization of the ACE2 protein in the ferret respiratory tract was investigated during the SARS-CoV-1 and SARS-CoV-2 outbreaks, with conflicting results. Using immunohistochemistry‚ van den Brand and colleagues detected ACE2 antigen in type-II pneumocytes, alveolar macrophages, tracheal, and bronchial and bronchiolar epithelial cells as well as endothelial and smooth muscle cells of pulmonary blood vessels of ferrets.^58^ In contrast, Lean and colleagues, using a validated antibody for ACE2, detected the antigen exclusively in the respiratory and olfactory epithelium of the nasal turbinate, but not in the tracheal, bronchial, bronchiolar, and alveolar epithelia.^28^ Thus, other factors besides ACE2 seem to determine cell tropism of these viruses in ferrets.

Interestingly, the clinical and pathological picture following SARS-CoV-2 infection differs markedly between ferrets and mink. Severe acute respiratory syndrome coronavirus 2 caused several outbreaks in mink farms in the United States, the Netherlands, Denmark, and Spain^31^ and mink can even transmit the virus back to humans and to other animals like cats.^41^ The clinical disease in mink is characterized by labored breathing and watery to mucoid nasal exudates of varying severity and death within 3 days in severe cases.^34^ The mortality can reach 9.8%. Macroscopically, the lungs are swollen, dark red, and fail to collapse. Histologically, alveolar septa are hyperemic, thickened due to fibrin extravasation and mild mononuclear cell infiltrates, and often lined by delicate hyaline membranes. Lesions are most prominent in areas adjacent to bronchi and include type-II pneumocyte hyperplasia, alveolar edema with abundant foamy macrophages and few neutrophils, and perivascular edema. Epithelial cells show loss of cilia, degeneration, and necrosis as well as syncytia formation.^34^ The reasons for the major differences in the clinical course and outcome of SARS-CoV-2 infection in ferrets and mink remain unclear but variations in expression patterns of ACE2 and neutrophilin-1 were suggested as possible host factors.^28,53^ In contrast, variations in the sequences of ferret and mink ACE2 proteins are most likely not responsible for the striking differences in their susceptibility to SARS-CoV-2 infection.^28,49^ When comparing the reports in these 2 species, one also has to consider the epidemiological aspects and circumstances of infection. The outbreaks in mink farms were associated with large numbers of animals kept in contact, which facilitated rapid spread and amplification of virus, most likely resulting in higher infection doses and/or repeated exposure of the animals. The situation cannot be directly compared with the controlled setting of experimental studies. Major outbreaks of SARS-CoV-2-induced disease with associated mortality have not been described in pet or hunting ferrets in natural conditions. In a recent report from Spain, the presence of SARS-CoV-2 RNA was investigated in 71 ferrets maintained for rabbit hunting.^18^ Viral RNA was detected in 6 out of 71 animals (8.4%), belonging to 4 out of 7 investigated groups. None of these animals showed any clinical signs and contact animals tested negative. Thus, ferrets can acquire natural SARS-CoV-2 infection, but prolonged virus circulation is not maintained in small ferret groups.

### Summary and Conclusion

Ferrets showed no or mild clinical signs, and SARS-CoV-2 viral replication occurred mainly in the upper respiratory tract.^15,62^ In contrast, SARS-CoV-1 infection induced more severe clinical signs in ferrets but fulminant pneumonia was not reliably reproducible^11,33,58^ and virus shedding was detected early in infection, which differed from the late peak of virus load and disease in humans.^25^ Mink infected with SARS-CoV-2 reliably develop COVID-19-like disease with viral replication and lesions in the upper and lower respiratory tract. Ferrets and mink are thus suitable models to study SARS-CoV-2 replication and transmission and could aid in the risk assessment of new virus variants of concern.^65^ Moreover, preventive and therapeutic intervention strategies such as compounds blocking virus entry, vaccines and antiviral drugs that aim to reduce viral replication, can be readily tested in these species.^25,44,53^ Mink and potentially also aged ferrets can be used to study the pathogenesis of lung lesions and associated therapeutic measures. However, mink have several drawbacks including their potential to give rise to new variants that may spill back to humans and therefore pose a potential risk for the human population and spread of SARS-CoV-2. Ferrets are well-established laboratory animals for various respiratory zoonotic pathogens with a wide range of analytical and molecular diagnostic tools. Various factors such as individual susceptibility,^24,48,52,62,65^ age,^24^ virus strain,^65^ infection route, and dose of the inoculum^35,48,52^ can influence the outcome of SARS-CoV-2 infection in ferrets. Hence, the use of ferrets or mink for betacoronavirus research has to be carefully considered according to the research question and aim of the study outcome.

## Supplemental Material

sj-pdf-1-vet-10.1177_03009858211071012 – Supplemental material for Ferrets are valuable models for SARS-CoV-2 research Click here for additional data file. Supplemental material, sj-pdf-1-vet-10.1177_03009858211071012 for Ferrets are valuable models for SARS-CoV-2 research by Malgorzata Ciurkiewicz, Federico Armando, Tom Schreiner, Nicole de Buhr, Veronika Pilchová, Vanessa Krupp-Buzimikic, Gülşah Gabriel, Maren von Köckritz-Blickwede, Wolfgang Baumgärtner, Claudia Schulz and Ingo Gerhauser in Veterinary Pathology
