# Supplementary material for: Ferrets are valuable models for SARS-CoV-2 research
Source: Vet Pathol. 2022 Jan 8;59(4):661–72. doi: 10.1177/03009858211071012 (PMC9207987; doi:10.1177/03009858211071012)
Supplement: sj-pdf-1-vet-10.1177_03009858211071012 – Supplemental material for Ferrets are valuable models for SARS-CoV-2 research [file sj-pdf-1-vet-10.1177_03009858211071012.pdf]

Malgorzata Ciurkiewicz, Federico Armando, Tom Schreiner, Nicole de Buhr, Veronika Pilchova, Vanessa Krupp-Buzimikic, Gülşah Gabriel, Maren von Köckritz-Blickwede, Wolfgang Baumgärtner, Claudia Schulz and Ingo Gerhauser

Suppl. Figures S1-S4. Lungs of control ferrets (**S1,** F2) and ferrets at 4 days post infection (dpi) (**S2,** F4), 7 dpi (**S3,** F8), and 21 dpi (**S4,** F12). No macroscopical lesions were present in control or SARS-CoV-2-infected ferrets. **Suppl. Figure S5.** Absolute and relative lung weights of control ferrets and infected ferrets at 4, 7 and 21 dpi. No statistically significant differences between control and infected animals were found using one-way ANOVA with Dunnett's multiple comparison tests (GraphPad Prism 9.0.0).

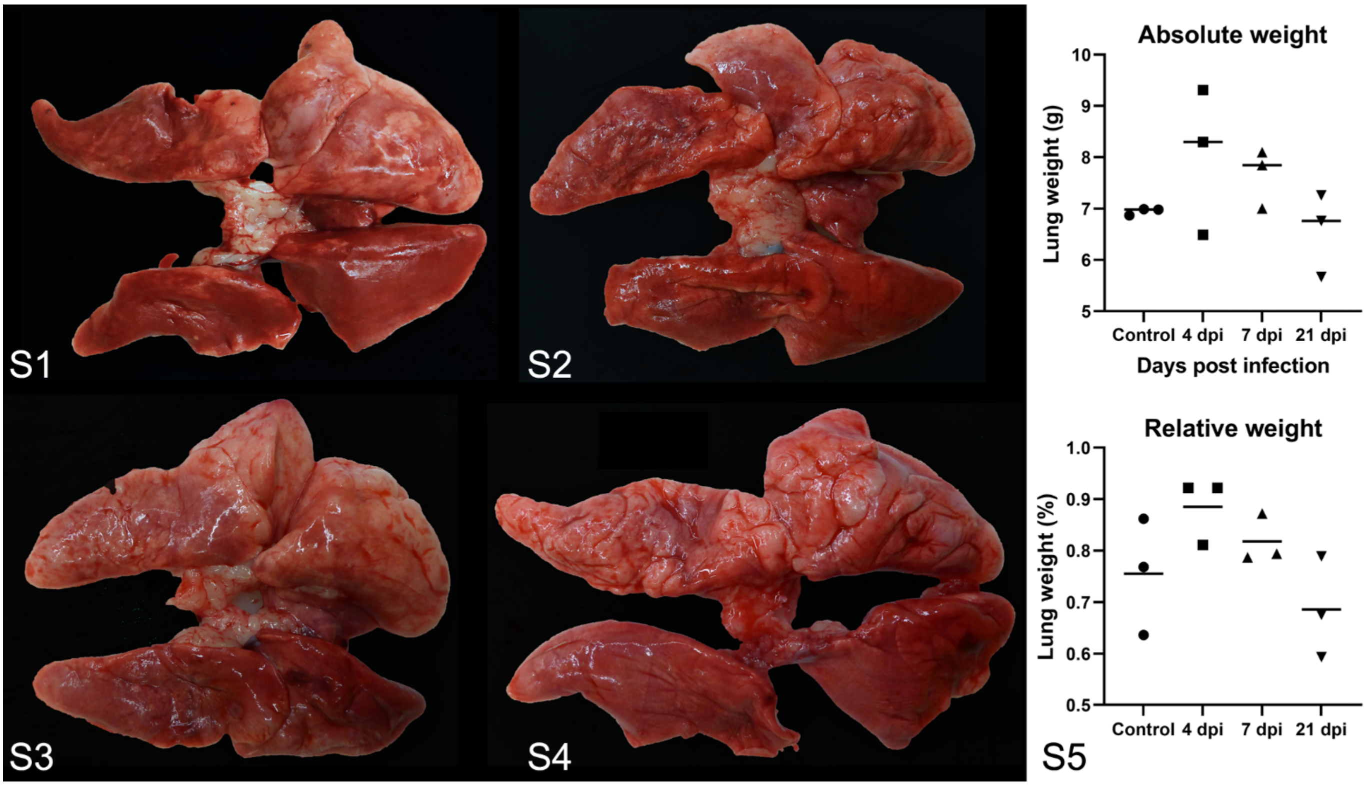

Suppl. Figures S6-S9. Lesions in the gastrointestinal tract and liver of control and SARS-CoV-2 infected ferrets. **Suppl. Figure S6.** Gastritis, control ferret (F1, HE). There is an infiltration of the mucosa with many lymphocytes, plasma cells and macrophages, a mild multifocal mineralization, and a large follicular structure below the gastric glands. Inset: higher magnification of the mucosa. **Suppl. Figure S7.** Enteritis, jejunum, SARS-CoV-2 infected ferret at 4 days post infection (dpi, F4, HE). The mucosa is infiltrated by many eosinophils as well as lymphocytes, plasma cells and macrophages. Inset: Higher magnification of inflammatory infiltrates in a villus. **Suppl. Figure S8.** Hepatitis, control ferret (F2, HE). The portal areas are infiltrated with many lymphocytes, plasma cells and macrophages. Inset: Higher magnification of inflammatory infiltrates. **Suppl. Figure S9.** Hepatitis and large follicular structures in the mucosa of the gallbladder, control ferret (F2, HE). Inset: Higher magnification.

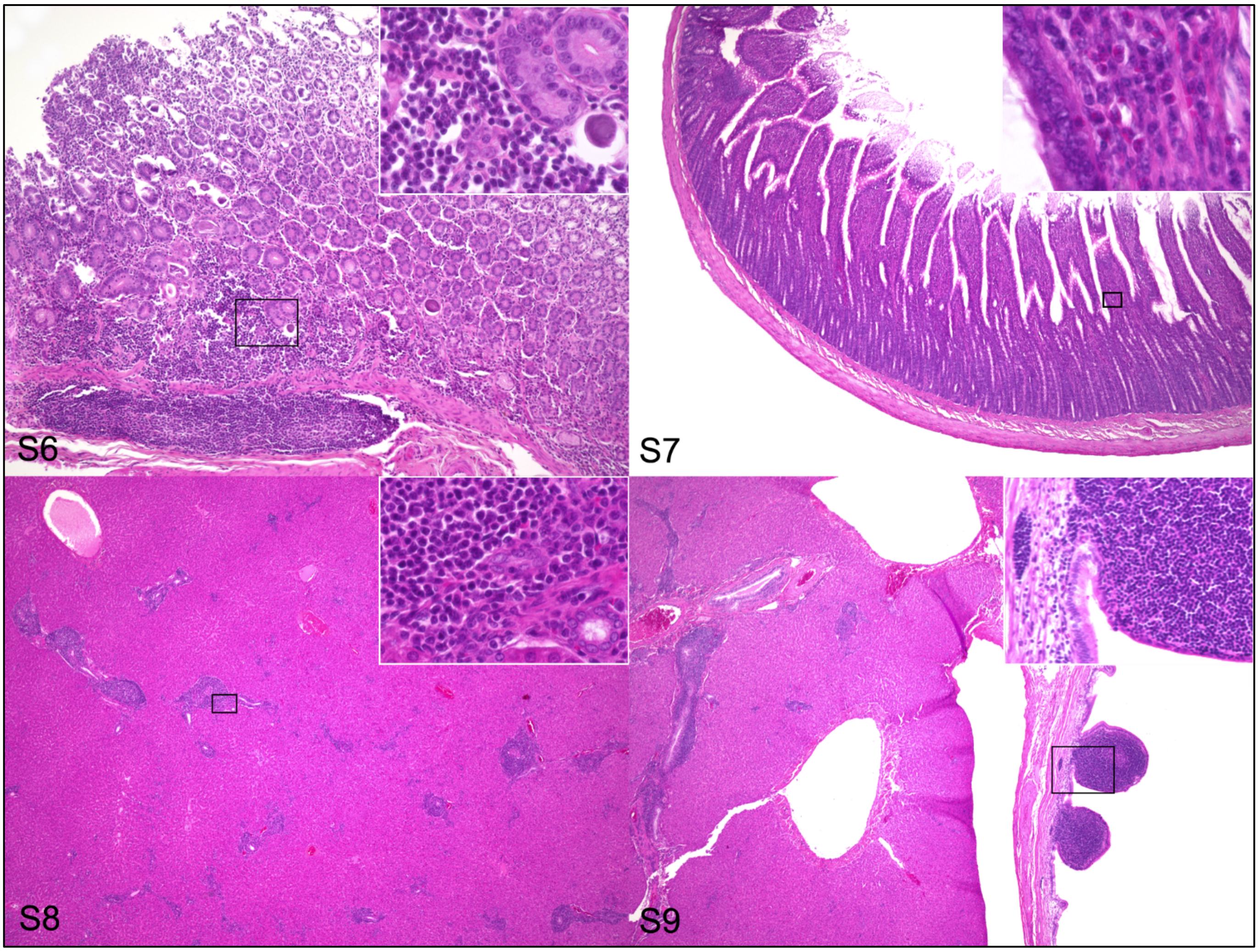

Suppl. Figures S10-S13. Incidental lesions in control and SARS-CoV-2 infected ferrets. **Suppl. Figure S10.** Meningitis, cerebrum, SARS-CoV-2 infected ferret, 7 days post infection (dpi, F9, HE). There is a perivascular infiltration of the meninges with few lymphocytes and macrophages. Inset: Higher magnification. **Suppl. Figure S11.** Endothelialitis and periarteritis, aorta, control ferret (F1, HE). The connective tissue adjacent to the aorta is infiltrated by several lymphocytes, plasma cells and macrophages, which can also be found below the endothelium (endothelialitis). Insets: Higher magnifications. **Suppl. Figure S12.** Sialoadenitis, SARS-CoV-2 infected ferret, 7 dpi (F7, HE). The parenchyma is infiltrated by several lymphocytes and macrophages. Inset: Higher magnification. **Suppl. Figure S13.** Endocarditis, SARS-CoV-2 infected ferret, 7 dpi (F9, HE). There is mild focal infiltration of the endocardium with lymphocytes and macrophages.

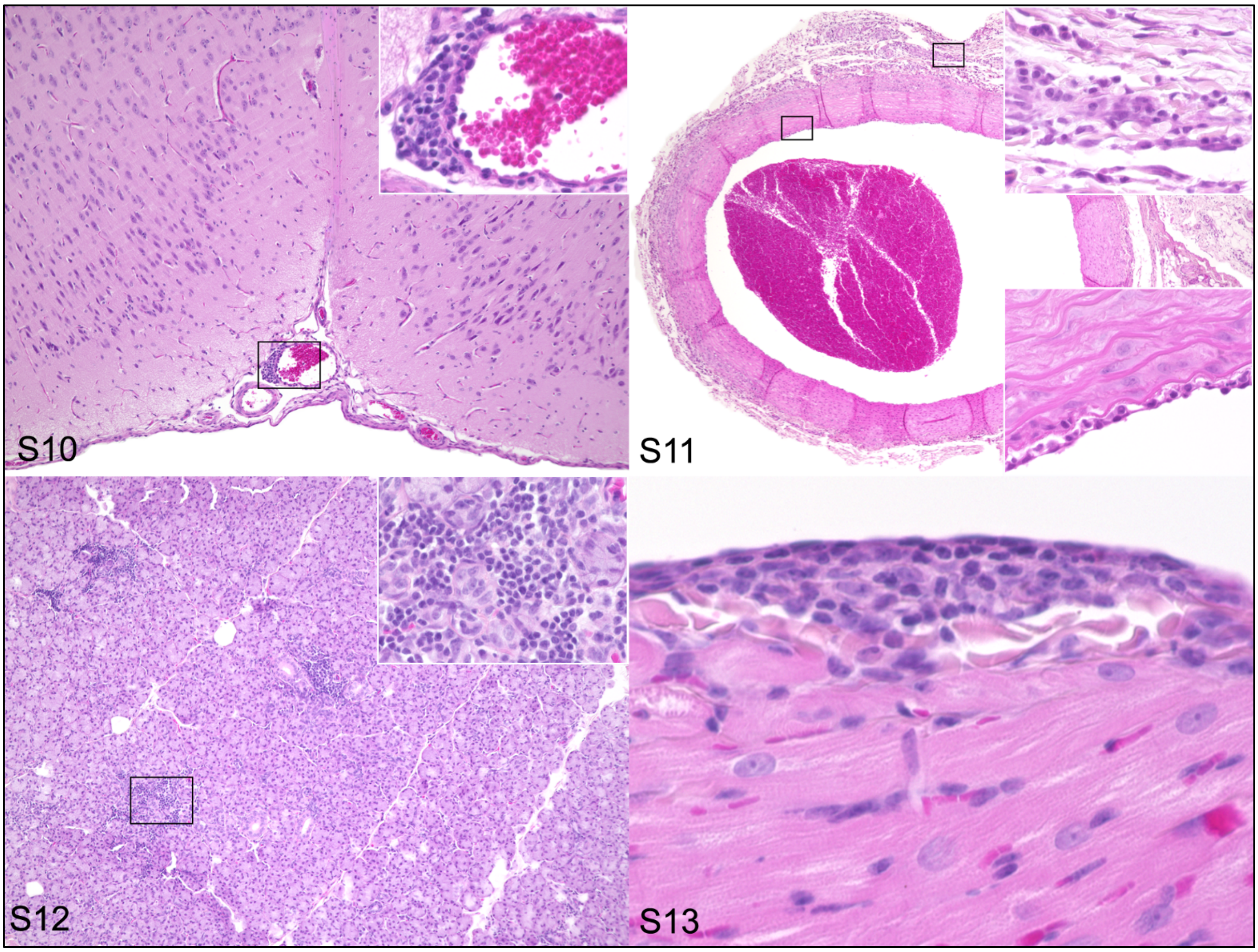

Supplemental Table S1. Clinical scoring of ferrets inoculated with SARS-CoV-2.

Clinical score parameter	Score
**Bodyweight**	
<5% weight loss	0
5-10% weight loss	1
11-15% weight loss	2
16-20% or acute weight loss > 20% in comparison to initial weight (≤ 24 h)	HEP
**Cardiovascular system**	
Normal	0
Pinched skin/mild enophthalmia (sunken eyes)/mild dehydration	1
Moderate to severe dehydration/moderate to severe enophthalmia (sunken eyes)	HEP
Animal cold, legs and abdominal skin dark blueish	HEP
**Coat/Skin condition**	
Trimmed	0
Coat slightly unkempt, mild small skin irritation	1
Slight piloerection or small lesions, moderate small skin irritation or inflammation	2
Marked piloerection or moderate (scale) lesions or skin inflammation, dirty body orifice	3
Severe/large scale lesions, severe skin inflammation	HEP
**Respiratory tract**	
Normal	0
Tachypnea (slight)	1
Tachypnea (moderate)	2
Dyspnea (marked) < 24 h	3
Dyspnea (marked) > 24 h or cold cyanotic skin	HEP
Mild serous ocular or nasal discharge	1
Moderate serous to purulent ocular of nasal discharge	2
Severe serous to purulent ocular of nasal discharge	3
**Environment**	
Normal	0
Loose stools or diarrhea	1
Hemorrhagic diarrhea	HEP
**Social behavior/general condition/locomotion**	
Normal (alert, curious, promptly stands up using four limbs equally)	0
Lack of grooming, mildly inactive (tired)	1
Not restricted in mobility, beginning kyphosis (hunched back), mildly inactive and depressed, reduced interaction with other animals/apathic	2
Hunched up back (kyphosis), lethargic, isolation or hyperactivity, aggression, self-mutilation, repeated turning towards painful region, vocal expression of pain	3
Severe kyphosis	HEP
Immobility/moribund	HEP
**Neurological scoring**	
Normal	0
Mild neurological signs (e.g. head tilt, beginning ataxia, paralysis)	3
Moderate to severe neurological signs, ataxia, paralysis, shaking, convulsions	HEP

Suppl. Table S1. Clinical scoring of ferrets inoculated with SARS-CoV-2 (continued).

Clinical score parameter	Score
**Rectal body temperature (°C)**	
37.8-40.0	0
> 40.0 but ≤ 40.5	1
> 40.5 but ≤ 41.5	2
> 41.5 or > 40.0 for > 5 days	3
> 41.5 or > 40.0 for > 5 days followed by rapid fall of temperature (< 37°C)	HEP

Actions

CS 1: review frequency of monitoring

CS 2: ≤ 12 h frequency of monitoring

CS 4: consult veterinarian

CS 2 in general condition for 2 consecutive days and CS ≥ 15 in other categories: implement humane endpoint

CS 3 in general condition for 2 consecutive days and CS ≥ 10 in other categories: implement humane endpoint

CS 6 > 48 h: implement humane endpoint

CS = 3 in > 2 categories: implement humane endpoint

HEP: implement humane endpoint

Supplemental Table S2. Procedures and samples collected during the animal experiment.

Dpi	Ferret ID	Procedures	Samples
-3	F1-F12	anesthesia	OPS*, FES*, blood*
-2	F1-F3	anesthesia, euthanasia	OPS*, FES*, blood*, BALF*, tissue**
0	F4-F12	Intratracheal SARS-CoV-2 infection	
2	F4-F12		OPS*, FES*
4	F4-F6	anesthesia, euthanasia	OPS*, FES*, blood*, BALF*, tissue**
4	F7-F12		OPS*, FES*
5	F7-F12		OPS*, FES*
7	F7-F9	anesthesia, euthanasia	OPS*, FES*, blood*, BALF*, tissue**
7	F10-F12		OPS*, FES*
9	F10-F12		OPS*, FES*
12	F10-F12		OPS*, FES*
14	F10-F12	anesthesia	OPS*, FES*, blood*
17	F10-F12		OPS*, FES*
21	F10-F12	anesthesia, euthanasia	OPS*, FES*, blood*, BALF*, tissue**

*PCR; **PCR, histology, immunohistochemistry, immunofluorescence, and scanning electron microscopy; BALF: bronchoalveolar lavage fluid; Dpi: day post infection; FES: fecal swab; blood, serum and whole blood; OPS: oropharyngeal swab; tissue: Tissue samples used for microscopic evaluation were taken from abdominal skin (including subcutaneous tissue), skeletal muscle (left biceps femoris), left eye (including third eyelid/conjunctiva), nasal mucosa, larynx, thyroid gland, trachea, lung, tracheobronchial lymph node, heart, aorta, thymus, retropharyngeal lymph node, mandibular lymph node, mandibular salivary gland, tongue, tonsil, esophagus, stomach, duodenum, jejunum, ileum, colon, rectum, mesenterial lymph node, spleen, liver, gallbladder, pancreas, adrenal gland, kidney, urinary bladder, brain (including olfactory bulb, cerebral cortex, hippocampus, thalamus, hypothalamus, midbrain, cerebellum and medulla oblongata), spinal cord (including cervical, thoracic and lumbar segments), peripheral nerve (left sciatic nerve) and bone marrow (additional samples taken for PCR analysis are underlined).

Suppl. Table S3: Primer and probe sequences used for the detection of partial SARS-CoV-2 RNA-dependent RNA polymerase (RdRp) (SARS2-IP4 assay) and internal control (EGFP assay) with real-time quantitative reverse transcription PCR.

Oligo name	Sequence of primer / probe (5' - 3')	References
SARS2-IP4-14059-F	GGT AAC TGG TAT GAT TTC G	1,2
SARS2-IP4-14146-R	CTG GTC AAG GTT AAT ATA GG	1,2
SARS2-IP4-14084-FAM	[FAM (Fluorescein)]-TCA TAC AAA CCA CGC CAG G-[BHQ-1]	1,2
EGFP1-F	GAC CAC TAC CAG CAG AAC AC	1,2,3
EGFP2-R	GAA CTC CAG CAG GAC CAT G	1,2,3
EGFP-Probe1-HEX	[HEX]-AGC ACC CAG TCC GCC CTG AGC A-[BHQ-1]	1,2,3

1: Hoffmann 2020 (https://www.dvg.net/fileadmin/Bilder/DVG/PDF/20-03-31-LA_RT-qPCR_SARS-CoV-2_IP4__AgPath_.pdf)

2: Jendrny P, Schulz C, Twele F, et al. Scent dog identification of samples from COVID-19 patients - a pilot study. *BMC Infect Dis*. 2020;20: 536.

3: Hoffmann B, Depner K, Schirrmeier H, Beer M. A universal heterologous internal control system for duplex real-time RT-PCR assays used in a detection system for pestiviruses. *J Virol Methods*. 2006;136: 200-209.

Supplemental Table S4. Clinical and virological results of the animal experiment.

Ferret ID	Group	Clinical signs	Oropha-ryngeal swab*	Fecal swab*	BALF**	Tissues**
F1	Control	/	-	-	-	-
F2	Control	/	-	-	-	-
F3	Control	/	-	-	-	-
F4	4 dpi	reduced general condition 1 dpi, mild weight loss 2 dpi	+ (2,4 dpi)	+ (2, 4 dpi)	+	+ in nasal mucosa, retroph. l.n., tongue, tonsil, trachea, lung, tracheobr. l.n., olfactory bulb, cerebrum, cerebellum, medulla obl., spinal cord, heart, liver, ileum, skel. musc.
F5	4 dpi	/	+ (2,4 dpi)	+ (4 dpi)	+	+ in third eyelid, nasal mucosa, retroph. l.n., tongue, tonsil, trachea, lung, tracheobr. l.n., olfactory bulb, cerebrum, cerebellum, medulla obl., spinal cord, heart, spleen, liver, ileum, colon, mes. l.n., kidney, skel. musc.
F6	4 dpi	/	+ (2,4 dpi)	+ (4 dpi)	+	+ in third eyelid, nasal mucosa, retroph. l.n., tongue, tonsil, trachea, lung, tracheobr. l.n., olfactory bulb, cerebrum, cerebellum, medulla obl., spinal cord, heart, spleen, liver, ileum, colon
F7	7 dpi	/	+ (2,4, 5, 7 dpi)	-	+	+ in retroph. l.n., tracheobr. l.n.
F8	7 dpi	mild weight loss 2 dpi	+ (2,4, 5, 7 dpi)	+ (2, 5 dpi)	+	+ in nasal mucosa, tongue, tonsil, tracheobr. l.n.
F9	7 dpi	/	+ (2,4, 5, 7 dpi)	+ (2, 7 dpi)	+	+ in nasal mucosa, retroph. l.n., tongue, tonsil, trachea, lung, tracheobr. l.n.
F10	21 dpi	/	+ (4 dpi)	-	-	-
F11	21 dpi	/	+ (2,4, 5, 7 dpi)	+ (2, 4, 5 dpi)	-	+ in retroph. l.n
F12	21 dpi	/	+ (2,4, 5, 7,9 dpi)	+ (2 dpi)	-	+ in tracheobr. l.n.

Supplemental Table S4. (continued).

+ detection of SARS-CoV-2 RNA using PCR; * samples collected during the experiment (0, 2, 4, 5, 7, 9, 12, 14, 17, 21 dpi); ** samples only collected at the experimental endpoints (4, 7, 21 dpi), listed are SARS-CoV-2 RNA-positive tissues; for full list of tested tissues see Suppl. Table S2. Abbreviations: tracheobr. l.n.: tracheobronchial lymph node; dpi: days post infection; medulla obl.: medulla oblongata; mes. l.n.: mesenteric lymph node; retroph. l.n.: retropharyngeal lymph node; skel. musc.: skeletal muscle.

Supplemental Table S5: PCR results of oropharyngeal swab, fecal swab and bronchoalveolar lavage fluid (BALF) samples from ferrets experimentally infected with SARS-CoV-2. Shown are the quantification cycles (Cq) values of each sample.

**Matrix**	**Ferret ID**	**Days post infection**							
		**0**	**2**	**4**	**5**	**7**	**9**	**12, 14, 17**	**21**
Oropharyngeal swab	F4	NC	**23.5**	**27.8**	NC	NC	NC	NC	NC
	F5	NC	**24.0**	**21.1**	NC	NC	NC	NC	NC
	F6	NC	**37.0**	**31.0**	NC	NC	NC	NC	NC
	F7	NC	**28.2**	**33.2**	**32.5**	**34.1**	NC	NC	NC
	F8	NC	**31.3**	**30.6**	**31.0**	**32.4**	NC	NC	NC
	F9	NC	**27.2**	**29.8**	**33.6**	**30.9**	NC	NC	NC
	F10	NC	NC	**37.7**	NC	NC	NC	NC	NC
	F11	NC	**28.8**	**28.8**	**25.8**	**32.6**	NC	NC	NC
	F12	NC	**28.6**	**24.4**	**25.8**	**25.6**	**30.6**	NC	NC
Fecal swab	F4	NC	**32.0**	**37.5**	NC	NC	NC	NC	NC
	F5	NC	NC	**31.5**	NC	NC	NC	NC	NC
	F6	NC	NC	**37.6**	NC	NC	NC	NC	NC
	F7	NC	NC	NC	NC	NC	NC	NC	NC
	F8	NC	**34.3**	NC	**36.7**	NC	NC	NC	NC
	F9	NC	**37.4**	NC	NC	**37.6**	NC	NC	NC
	F10	NC	NC	NC	NC	NC	NC	NC	NC
	F11	NC	**34.7**	**34.7**	**36.0**	NC	NC	NC	NC
	F12	NC	**36.4**	NC	NC	NC	NC	NC	NC
BALF	F4	ND	ND	**23.0**	ND	ND	ND	ND	ND
	F5	ND	ND	**21.2**	ND	ND	ND	ND	ND
	F6	ND	ND	**25.1**	ND	ND	ND	ND	ND
	F7	ND	ND	ND	ND	**37.5**	ND	ND	ND
	F8	ND	ND	ND	ND	**33.0**	ND	ND	ND
	F9	ND	ND	ND	ND	**29.9**	ND	ND	ND
	F10	ND	ND	ND	ND	ND	ND	ND	NC
	F11	ND	ND	ND	ND	ND	ND	ND	NC
	F12	ND	ND	ND	ND	ND	ND	ND	NC

NC, No Cq (threshold at Cq 40); ND, not done

Supplemental Table S6: PCR results of tissue samples from ferrets experimentally infected with SARS-CoV-2. Shown are the copies/g tissue of each sample (see also Figure 2).

**Days post infection**		**4**			**7**			**21**	
**Ferret ID**	**F4**	**F5**	**F6**	**F7**	**F8**	**F9**	**F10**	**F11**	**F12**
**Third eyelid**	NC	**2736**	**4086**	NC	NC	NC	NC	NC	NC
**Nasal mucosa**	**4405595640**	**1023081478**	**4533066**	NC	**120571**	**2411792**	NC	NC	NC
**Retropharyngeal l.n.**	**14302926**	**4761405**	**58518**	**87440**	NC	**9531**	NC	**141**	NC
**Tongue**	**24972531**	**83481270**	**678165**	NC	**18594**	**13358**	NC	NC	NC
**Tonsil**	**88212644**	**687506593**	**992808**	NC	**5921**	**82393**	NC	NC	NC
**Trachea**	**2824536**	**6969824**	**287657**	NC	NC	**5343**	NC	NC	NC
**Lung caudal**	**218060**	**1467763**	**51026**	NC	NC	**2922**	NC	NC	NC
**Lung cranial**	**1393872**	**2355400**	**845**	NC	NC	NC	NC	NC	NC
**Tracheobronchial l.n.**	**3444227**	**28070222**	**26912**	**150195**	**5770**	**569**	NC	NC	**30**
**Olfactory bulb**	**179545008**	**94798**	**668963**	NC	NC	NC	NC	NC	NC
**Cerebrum**	**99478963**	**8032**	**198625**	NC	NC	NC	NC	NC	NC
**Cerebellum**	**1851287**	**220659**	**1425499**	NC	NC	NC	NC	NC	NC
**Medulla oblongata**	**2174229**	**330429**	**10404518**	NC	NC	NC	NC	NC	NC
**Spinal cord**	**26330**	**17074**	**398726**	NC	NC	NC	NC	NC	NC
**Heart**	**2970**	**124770**	**2156**	NC	NC	NC	NC	NC	NC
**Spleen**	NC	**958155**	**956**	NC	NC	NC	NC	NC	NC
**Liver**	**124**	**56329**	**42206**	NC	NC	NC	NC	NC	NC
**Ileum**	**629**	**2725321**	**21452**	NC	NC	NC	NC	NC	NC
**Colon**	NC	**34989**	**128**	NC	NC	NC	NC	NC	NC
**Mesenterial l.n.**	NC	**4159778**	NC	NC	NC	NC	NC	NC	NC
**Kidney**	NC	**32191250**	NC	NC	NC	NC	NC	NC	NC
**Skeletal muscle**	**4856**	**1888448**	NC	NC	NC	NC	NC	NC	NC

NC, No Cq (threshold at Cq 40)

Supplemental Table S7: Inflammatory lesions in the respiratory tract.

Ferret ID	Group	Nose	Trachea	Bronchi/ Bronchioli	Alveoli	Lung vessels
F1	Control	-	-	+	-	-
F2	Control	-	-	+	-	-
F3	Control	-	-	+	-	-
F4	4 dpi	+	+	+/++	-	-
F5	4 dpi	++	++	+	+	+
F6	4 dpi	+	+	+/++	-	-
F7	7 dpi	+	-	+/++	+	+/++
F8	7 dpi	-	-	+	-	-
F9	7 dpi	+	-	+	-	-
F10	21 dpi	-	-	+	-	-
F11	21 dpi	+	-	+/++	+	+/++
F12	21 dpi	+	-	-	-	-

- no inflammation; **+** mild inflammation; **++** moderate inflammation; dpi: days post infection.

Supplemental Table S8: Inflammatory lesions in extrarespiratory organs.

Ferret ID	Group	Organs
F1	Control	aorta **+/++**; stomach **+/++**; intestine **+**; liver **++**; kidney **+**
F2	Control	conjunctiva **++**; stomach **+/++**; intestine **+/++**; liver **++**
F3	Control	aorta **+**; liver **+**
F4	4 dpi	conjunctiva **+/++**; stomach **+**; intestine **+/++**; liver **+**
F5	4 dpi	skin **++**; stomach **+**; intestine **+**; liver **+/++**; kidney **++**; meninx **+**
F6	4 dpi	stomach **+**; intestine **+/++**; liver **+/++**
F7	7 dpi	conjunctiva **++**; skin **+**; salivary gland **+/++**; stomach **+/++**; intestine **+/++**; liver **+/++**; meninx **+**
F8	7 dpi	pharynx **++**; stomach **+**; intestine **+/++**; liver **+/++**
F9	7 dpi	skin **+**; heart **+**; stomach **++**; intestine **+/++**; liver **++/+++**; kidney **+**; meninx **+**
F10	21 dpi	stomach **+/++**; intestine **+/++**; liver **+**; kidney **+**
F11	21 dpi	conjunctiva **+/++**; stomach **++**; intestine **+/++**; liver **+/++**
F12	21 dpi	intestine **+/++**; liver **+/++**

+ mild inflammation; **++** moderate inflammation; **+++** severe inflammation; dpi: days post infection.

Supplemental Table S9: Studies investigating ferrets experimentally infected with SARS-CoV-2.

Author	Study design	Clinical signs	Virology	Histology	Molecular biology
Everett et al. 2021	Animals:<br>12 female ferrets 5 m<br>SARS-CoV-2:<br>2/Australia/VIC 01/2020<br>Dose:<br>1.2×10^6 $TCID_{50}$<br>Route:<br>intranasally	mild or none<br><br>fever<br>no weight loss	Nasal washes (RNA+):<br>high levels in 4/12 ferrets (33%), peak 4-6 dpi, no detection 14 dpi<br>Throat swabs (RNA+):<br>10-fold lower than nasal washes<br>Organs (RNA+):<br>high levels: nose, soft palate, tonsil, tongue, larynx (peak 7 dpi)<br>low levels: oesophagus, stomach (ingestion of viral particles?)<br>no viral RNA: spleen, liver, heart, trachea, cranial lung lobes, serum	Nasal cavity:<br>nasal turbinates with intact mucosal epithelial cells and rare presence of intra-epithelial neutrophils and apoptotic bodies<br>Lung:<br>variably congested with occasional bronchiolitis<br>Liver:<br>Moderate periportal lymphoplasmacytic hepatitis	IHC (antigen+):<br>nose: respiratory and olfactory epithelium (olfactory neuronal cells, sustentacular cells)<br>lung and olfactory bulb: negative<br><br>ISH (RNA+):<br>nose: respiratory and olfactory mucosa (olfactory neuronal cells, sustentacular cells)<br>olfactory bulb: negative
Kim et al. 2020	Animals:<br>24 ferrets<br>SARS-CoV-2:<br>NMC-nCoV02<br>Dose:<br>$10^{5.5}$ $TCID_{50}$<br>Route:<br>intranasally	mild<br><br>2-6 dpi:<br>occasional coughs<br>reduced activity<br>2-8 dpi:<br>fever<br>no weight loss	Nasal washes (RNA+):<br>peak 4 dpi, persisting 4-8 dpi, below detection limit 10 dpi<br><br>Organs (RNA+):<br>8 dpi: nose, trachea, lung, kidney, intestine	Lung:<br>acute bronchiolitis; increased immune infiltration and cell debris in alveolar walls, bronchial epithelium and lumen	IHC (antigen+):<br>nose, trachea, lung, intestine

Supplemental Table S9 (continued)**:** Studies investigating ferrets experimentally infected with SARS-CoV-2.

Author	Study design	Clinical signs	Virology	Histology	Molecular biology
Kim et al. 2021<br><br>(Preprint)	Animals:<br>G1: 9 female ferrets ≤ 6 m<br>G2: 9 female ferrets 12-24 m<br>G3: 9 female ferrets ≥ 36 m<br>SARS-CoV-2: NMC-nCoV02<br>Dose: $10^{5.8}$ $TCID_{50}$<br>Route: Intranasally	mild to moderate<br><br>G1: no fever, < 5% weight loss<br>G2: fever 2-6 dpi, ≤ 10% weight loss with rapid recovery<br>G3: fever 2-10 dpi, ≤ 10% weight loss	Nasal wash (infectious virus+):<br>G1: 2-6 dpi<br>G2: 2-8 dpi<br>G3: 2-8 dpi (higher viral titers)<br>Fecal specimens (RNA+):<br>G1: 2-4 dpi<br>G2: 2-8 dpi<br>G3: 2-8 dpi (higher copy numbers)<br>Nose & lung (viral titers): G1<G2<G3	G1&G2: only mild to moderate inflammation<br>G3: > 50% lung damage; widened, edematous, and congested alveolar septa (5 dpi)	ISH (RNA+):<br>more infected cells in G2&G3 compared to G1<br><br>RNA-seq analysis (genes upregulated):<br>G1: tissue remodeling (2 dpi)<br>G3: interferon response, innate immune response, B cell response, T cell response, chemokines (2 dpi)
Monchatre-Leroy et al. 2021	Animals:<br>15 ferrets 10 m<br>SARS-CoV-2: UCN19<br>Dose: 2×10^3 PFU<br>Route: intranasally	mild or none<br><br>7-8 dpi: lethargy<br>7-14 dpi: snoring<br><br>no fever<br>no weight loss	Organs (RNA+):<br>2 dpi: nose, tonsils, trachea, lung, liver, spleen, large intestine, kidney, brain<br>14 dpi: nose, tonsils, medulla oblongata<br>urine samples: negative<br>Infectious virus pos.: nose, nasal washes; NOT lung<br>Neutralizing antibodies: 7-10 dpi	Lung:<br>Non-infected ferrets: minimal to mild peribronchiolar and perivascular cuffing<br>Infected ferrets (2-14 dpi): mild bronchiolitis with leukocytes in bronchiolar lumina; moderate perivascular and peribronchiolar cuffing (mainly mononuclear cells); small consolidated areas (macrophages and lymphocytes within parenchyma)<br>Tonsils and trachea: no change	ISH (RNA+):<br>few scattered cells within alveolar walls and trachea in 1/3 ferrets at 2 dpi (not related to any histopathological lesion)

Supplemental Table S9 (continued)**:** Studies investigating ferrets experimentally infected with SARS-CoV-2.

Author	Study design	Clinical signs	Virology	Histology	Molecular biology
Ryan et al. 2021	SARS-CoV-2:<br>Victoria/1/2020<br><br>Dose:<br>5 × 10^6 PFU (high)<br>5 × 10^4 PFU (medium)<br>5 × 10^2 PFU (low)<br><br>Route:<br>intranasally	mild<br><br>reduced activity ruffled fur<br><br>no fever<br>no weight loss, transient failure to gain weight	Nasal washes (RNA+):<br>1/6 ferrets (low); 6/6 ferrets (medium, high), peak 2-6 dpi; until 18 dpi<br>BAL (RNA+): 3-7 dpi (low, medium, high)<br>Organs (RNA+): nose, tonsil, trachea, lung<br>Subgenomic RNA+:<br>nasal washes and throat swabs (medium, high); BAL (high)<br>Neutralizing antibodies:<br>8-14 dpi (medium, high)	Nasal cavity (3-7 dpi):<br>mild epithelial necrosis, inflammation<br>Lung (3-21 dpi; medium, high):<br>mild suppurative partly lympho-histiocytic bronchopneu-monia in 5-15% of lung; mild necrosis of bronchiolar epithelial cells; neutrophils and mononuclear cells in bronchiolar luminae; mild interstitial pneumonia; mild BALT hyperplasia; mild type II pneumocyte proliferation<br>Liver: lymphohistioplasmacytic portal hepatitis (more severe in infected animals)	ISH (RNA+, medium, high):<br>nose: abundant epithelial cells (3 dpi); scattered cells (5, 7 dpi)<br>larynx and trachea: very few scattered epithelial cells (3-7 dpi)<br>small and large intestine: occasional epithelial enterocytes and goblet cells (without histo-pathological lesions)
Schlottau et al. 2020	Animals:<br>12 female ferrets 9-12 m<br>SARS-CoV-2:<br>2019_nCoV Muc-IMB-1<br>Dose:<br>10^5 $TCID_{50}$<br>Route:<br>intranasally	none<br><br>no fever<br>no weight loss	Organs (RNA+):<br>4 dpi: muscle, skin, nose, trachea, lung, lung lymph node, colon<br>21 dpi (very weak):<br>brain (Cq 37.78), colon (Cq 37.47)<br>Antibodies:<br>> 8 dpi	Nasal cavity:<br>4 dpi: mild rhinitis with epithelial degeneration, necrosis, intraluminal cellular debris<br>8, 12 dpi: more severe rhinitis<br>21 dpi: rhinitis mild or absent<br>Lung:<br>slight alveolar histiocytosis	IHC (antigen+):<br>nose: respiratory and olfactory epithelium (4-8 dpi)<br>lung: negative

Supplemental Table S9 (continued)**:** Studies investigating ferrets experimentally infected with SARS-CoV-2.

Author	Study design	Clinical signs	Virology	Histology	Molecular biology
Shi et al. 2020	Animals:<br>10 ferrets<br>SARS-CoV-2:<br>2/F13/environment/2020/Wuhan (F13-E)<br>2/CTan/human/2020/Wuhan (CTan-H)<br>Dose:<br>10^5 PFU<br>Route:<br>intranasally	mild or none<br><br>10 dpi:<br>fever<br><br>12 dpi:<br>loss of appetite	Organs (RNA+ and infectious virus+):<br>4 dpi: nose, soft palate, tonsils; NOT trachea, lung, heart, liver, spleen, kidneys, pancreas, small intestine, brain<br>viral RNA higher in nasal than rectal swabs; antibody titers 13 dpi notably lower than 20 dpi	Lung (13 dpi):<br>severe lymphoplasmacytic perivasculitis and vasculitis; increased numbers of type II pneumocytes, macrophages, neutrophils in alveolar septa and lumina; mild peribronchitis	ND
Shi et al. 2020	Animals:<br>8 ferrets<br>SARS-CoV-2:<br>CTan-H<br>Dose:<br>10^5 PFU<br>Route:<br>intratracheally!	ND	Organs (RNA+):<br>2,4 dpi: nose, soft palate<br>8 dpi: nose, soft palate, tonsils, trachea<br>14 dpi: not present	ND	ND
Zaeck et al. 2021<br><br>Optically Clear Tissue Samples!	see Schlottau et al. 2020<br><br>Animals:<br>1 infected ferret euthanized at 4 dpi and 1 naïve animal	see Schlottau et al. 2020	see Schlottau et al. 2020	see Schlottau et al. 2020<br><br>Light Sheet Microscopy (antigen+):<br>nose: concha nasalis dorsalis et ventralis<br>lung: above epithelial cells	see Schlottau et al. 2020<br><br>Confocal Laser-Scanning Microscopy (antigen+):<br>nose: ciliated & non-ciliated cells

Supplemental Table S9 (continued)**:** Studies investigating ferrets experimentally infected with SARS-CoV-2.

Author	Study design	Clinical signs	Virology	Histology	Molecular biology
Zhou et al. 2021	<u>Animals:</u><br>4 female ferrets<br>8 male ferrets<br>4-18 m<br><u>SARS-CoV-2:</u><br>USA-WA1/2020 (S-614D)<br>Massachusetts/ VPT1/2020 (S-614G)<br><u>Dose:</u><br>$10^{5.4}$ $TCID_{50}$<br><u>Route:</u><br>Intranasally	mild<br><br>no weight loss	direct “one-to-one" transmission experiment: SARS-CoV-$2^{S\text{-}614G}$ became the dominant variant in 5/6 ferrets<br>SARS-CoV-2 transmission in 4/6 ferrets (mainly SARS-CoV-$2^{S\text{-}614G}$)<br><u>Nasal washes (RNA+):</u><br>6/6 donor ferrets 2 dpi<br>4/6 contact ferrets 2 days after contact	ND	ND

dpi, days post infection; IHC, immunohistochemistry; ISH, in-situ hybridization; m, months; ND, not done

Supplemental Table S10: Studies investigating ferrets experimentally infected with SARS-CoV-1.

Author	Study design	Clinical signs	Virology	Histology	Molecular biology
Chu et al. 2008	Animals:<br>42 ferrets<br>SARS-CoV-1:<br>Tor2<br>Dose:<br>10^3 $TCID_{50}$<br>Route:<br>intranasally	mild<br><br>2 and 6 dpi:<br>fever<br>Within 29 dpi:<br>17/42 animals with sneezing<br>5/42 animals with diarrhea	Lung:<br>2 dpi: 10^3 $TCID_{50}$ U/mL<br>5 dpi: 10^5 $TCID_{50}$ U/mL<br>Nasal turbinates:<br>2 dpi: 10^7 $TCID_{50}$ U/mL<br>7 dpi: 10^6 $TCID_{50}$ U/mL<br>Nasal & pharyngeal swabs, nasal turbinates & lung homogenates (RNA+): 2-7 dpi	Lung (7 dpi):<br>mild lymphohistiocytic bronchointerstitial pneumonia; mucus and neutrophils in bronchial lumina (only most severely affected animals)	ND
Czub et al. 2005	Animals:<br>12 ferrets<br>SARS-CoV-1:<br>Tor2<br>Dose:<br>10^6 PFU<br>Route:<br>intranasally	none<br><br><br>no fever<br>no weight loss	Feces (RNA+):<br>1-6 dpi<br>Pharyngeal swabs (RNA+):<br>1-22 dpi; 9/12 positive 10 dpi, 2/12 positive 22 dpi<br>Blood (RNA+):<br>8-22 dpi	Lung: ND<br>Liver (27-29 dpi):<br>periportal and panlobular hepatitis; more severe liver cell necrosis in vaccinated animals (recombinant modified vaccinia Ankara (MVA) expressing SARS-CoV S and N proteins)	ND
van den Brand et al. 2008	Animals:<br>4 ferrets<br>SARS-CoV-1:<br>HKU39849<br>Dose:<br>10^6 $TCID_{50}$<br>Route:<br>intratracheally	mild<br><br>2-4 dpi :<br>lethargy<br><br>4 dpi:<br>1 ferret died	ND	Lung (4 dpi): multifocal mild to severe exudative diffuse alveolar damage (DAD); moderate thickening of the alveolar septa with epithelial necrosis; multifocal type II pneumocyte hyperplasia<br>Liver (4 dpi): mild diffuse lipidosis<br>Spleen, trachea, bronchial lymph nodes (4 dpi): mild lymphoid hyperplasia	IHC (Antigen+; 4 dpi):<br>limited to respiratory tract (mainly type II rather than type I pneumocytes)

dpi, days post infection; IHC, immunohistochemistry; ISH, in-situ hybridization; m, months; ND, not done
